# A falcotentorial dural arteriovenous fistula presented as carotid cavernous fistula clinically treated by transarterial embolization: case report

**DOI:** 10.1186/s41016-022-00309-w

**Published:** 2022-12-14

**Authors:** Yuan Shi, Peixi Liu, Yingtao Liu, Kai Quan, Yanlong Tian, Wei Zhu

**Affiliations:** 1 Department of Neurosurgery, Huashan Hospital, Shanghai Medical College, Fudan University, Shanghai, China; 2 National Center for Neurological Disorders, Shanghai, China; 3 Shanghai Key Laboratory of Brain Function and Restoration and Neural Regeneration, Shanghai, China; 4 Neurosurgical Institute of Fudan University, Shanghai, China; 5 Shanghai Clinical Medical Center of Neurosurgery, Shanghai, China; 6 Department of Radiology, Huashan Hospital, Shanghai Medical College, Fudan University, Shanghai, China

**Keywords:** Dural arteriovenous fistulas, Falcotentorial DAVF, Ocular symptoms, Case report

## Abstract

**Background:**

Dural arteriovenous fistulas (DAVF) represent almost 10–15% of intracranial malformations that cause intracranial hemorrhage and focal neurological deficits. Seldom tentorial DAVF cases present with ocular manifestations initially, which occur frequently in carotid–cavernous fistula (CCF) and cavernous sinus DAVF (CS DAVF).

**Case presentation:**

We report an unusual falcotentorial DAVF case draining via the superior and inferior ophthalmic veins that caused left-side increased intraocular pressure. The patient’s chief complaint was swelling on the left side, pain and conjunctival congestion. He received endovascular embolization via a transarterial approach, and postoperative angiography demonstrated that the falcotentorial DAVF was occluded completely.

**Conclusion:**

Except for CCF and CS DAVF, some specific subtypes of DAVF should be considered if the patient initially presents with ocular symptoms. Differential diagnosis and definitive treatment are mandatory to avoid a delayed diagnosis and irreversible symptoms.

## Background

Dural arteriovenous fistulas (DAVF), an abnormal communication within the dural leaflets, represent almost 10–15% of intracranial malformations that can cause life-threatening hemorrhage or progressive focal neurological deficits [1, 2]. DAVFs in varied locations present with symptoms based on venous drainage patterns and sectoral congestion. Tentorial DAVF is an aggressive intracranial vascular lesion causing progressive neurological deficits. The clinical presentations of carotid–cavernous fistula (CCF) is usually associated with ocular manifestations, including chemosis, pulsatile exophthalmos, visual impairment, and ocular motility disturbances caused by anterior venous drainage [2], which are uncommon in other DAVF cases, especially in tentorial DAVF cases.

The falcotentorial DAVFs present as CCF are rarely encountered in clinical practice. We reported and illustrated the treatment of a falcotentorial DAVF with ocular symptoms, which is similar to left-side CCF.

## Case presentation

A 32-year-old male patient presented with left eye swelling, slight pain and conjunctival congestion in the past 6 months. The patient was diagnosed with left-side conjunctivitis and received anti-infection therapy in a local hospital. However, his symptoms were not relieved.

The patient was admitted to our department with a primary diagnosis of left-side CCF. Ophthalmologic examination showed no pathological findings. There was no decline in vision (Vod: 1.0; Vos: 1.0), visual acuity or slightly increased intraocular pressure. The visual field and ocular motility were normal. The results of ophthalmoscopic examination were negative. No significant evidence of neurological deficits or raised intracranial pressure was observed. The patient denied a history of head trauma and was not on other specific medications. There was a significant family history, and a comprehensive review of the systems was also noncontributory.

**Fig. 1:**
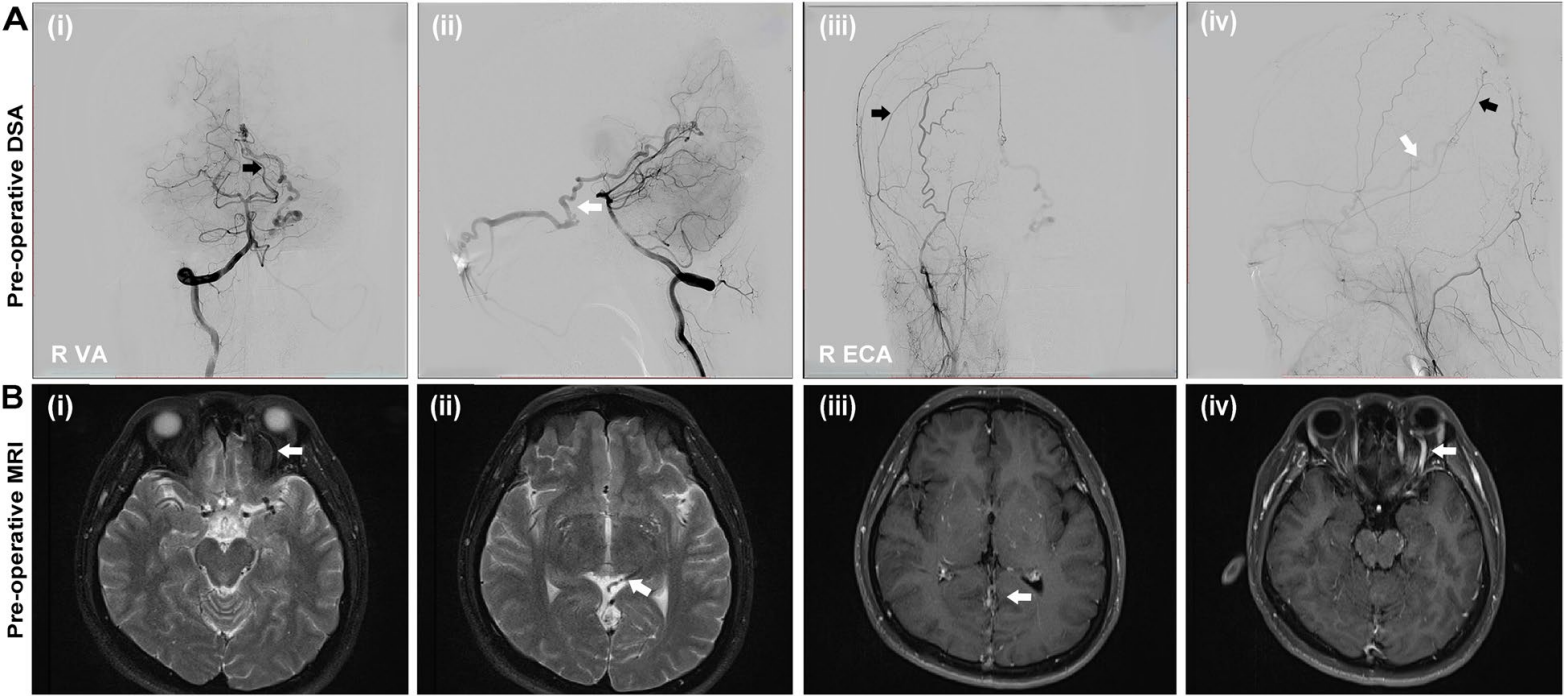
**A** Preoperative selective cerebral DSA showed the presence of falcotentorial DAVF fed from the left posterior cerebral artery and branches of the right middle meningeal artery (black arrow). Vein draining into the cavernous sinus and superior and inferior ophthalmic veins (white arrow). **B** Preoperative MR (i–ii T2 WI) and enhanced MR (iii–iv) showed an enlarged left side superior ophthalmic vein (white arrow), and a flow void effect was observed (i–ii)

Preoperative MR showed an enlarged left side superior ophthalmic vein, and a flow void effect was observed on T2WI (Fig. 1B). These MRI findings suggested the existence of intracranial vascular abnormalities. Superselective digital subtraction angiography (DSA) was performed, and access was obtained to the right femoral artery. Angiography confirmed the presence of a falcotentorial DAVF with feeding arteries arising from the left posterior cerebral artery (PCA) dural branches and branches of the right middle meningeal artery (MMA). The draining vein was an anonymous variant vein. The abnormally enlarged draining vein went along the cerebellum tentorium and finally into the left cavernous sinus. Cavernous sinus hypertension induced retrograde flow of the superior and inferior ophthalmic veins, which contributed to ophthalmic congestive symptoms. (Fig. 1A).

**Fig. 2:**
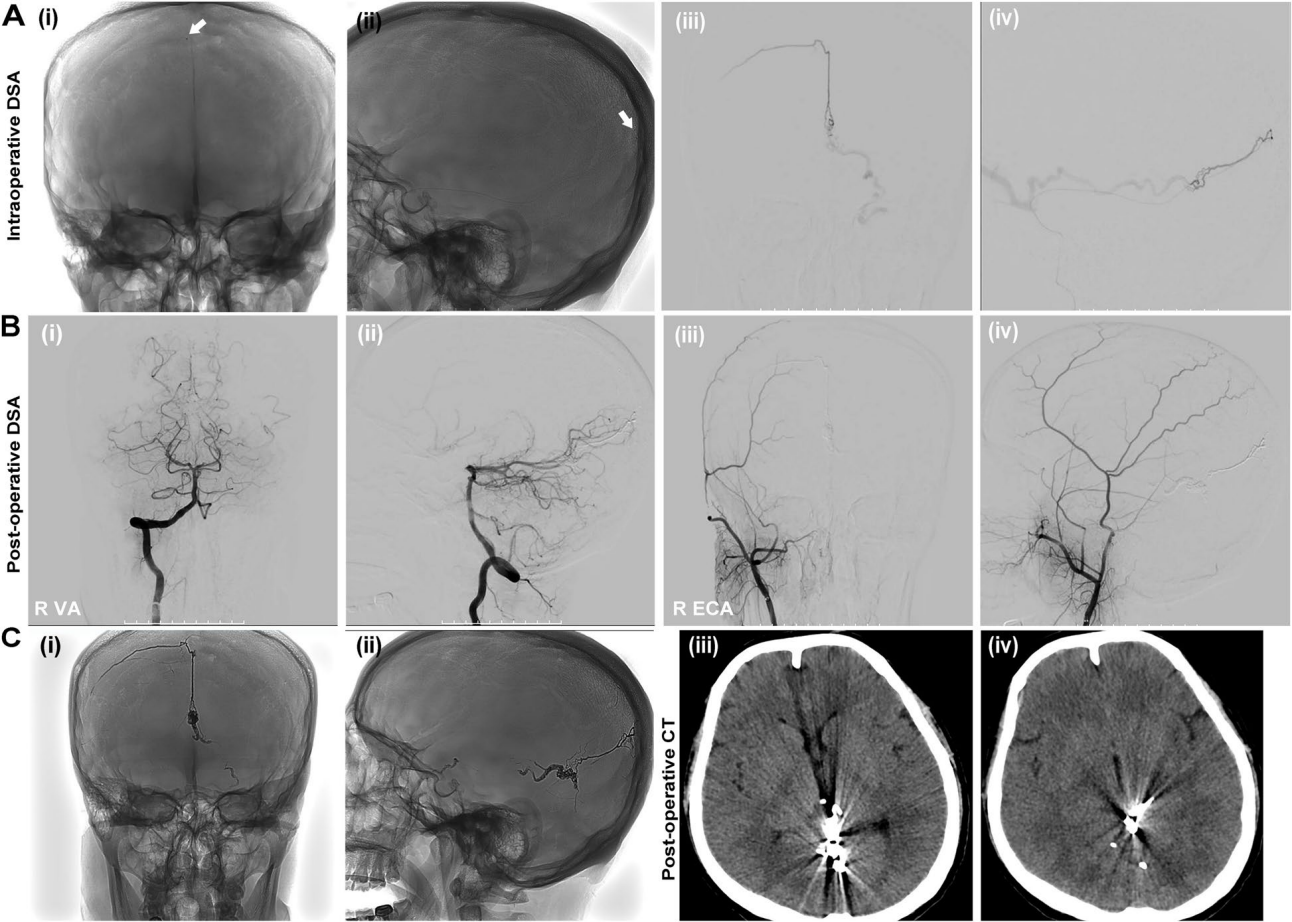
**A** Intraoperative angiographic imaging showed the position of the microcatheter tip before Onyx injection. (i–ii, white arrow). Angiography was performed via microcatheter (iii, iv). **B** Post-embolization angiography demonstrated that the DAVF was occluded completely without nontargeted vessel embolization. **C** Penetration and solidification of Onyx after embolization (i, ii). A postoperative CT scan showed no new cerebral infarction (iii, iv)

The patient decided to receive endovascular therapy. After general anesthesia, a 6-Fr Envoy guiding catheter (Codman, Miami Lakes, FL, USA) was positioned within the right external carotid artery. Then, a Marathon flow-directed microcatheter (ev3 Endovascular; Medtronic, Minneapolis, MN, USA) was advanced and placed at the posterior branch of the right MMA, and the tip of the microcatheter was close to the cerebral falx (Fig. 2A(i–ii)). Onyx-18 (ev3 Endovascular; Medtronic, Minneapolis, MN, USA) was injected carefully and allowed to diffuse to occlude fistulous connections, feeding arteries and partial draining veins (Fig. 2A(iii–iv)). Postoperative angiography demonstrated that the falcotentorial DAVF was occluded completely (Fig. 2B). The patient received a head CT scan on the first postoperative day, and no new cerebral infarction was observed (Fig. 2C).

### Outcome and follow-up

**Fig. 3:**
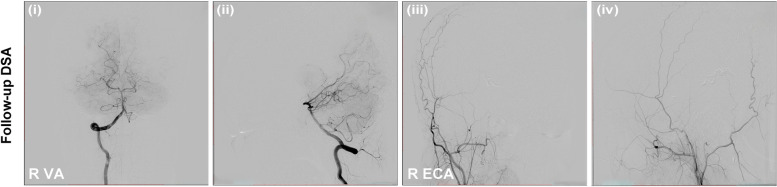
Nine-month follow-up cerebral angiography demonstrated no evidence of DAVF recurrence

On post-embolization day 1, the patient felt the ocular symptoms were completely relieved. Nine-month follow-up cerebral angiography demonstrated no evidence of DAVF recurrence, and no procedure-related complications were observed (Fig. 3).

## Discussion

DAVF is a rare type of acquired vascular malformation of the intracranial venous system. The DAVF can occur anywhere within dura maters, and most of them are located at cavernous and transverse-sigmoid sinuses. Symptoms and signs of DAVF depend on its draining patterns. Although the pathophysiology of DAVF remains controversial, it has been suggested that intracranial venous hypertension represents one key factor in the etiology of DAVF [3, 4]. The natural history and therapeutic indications of DAVF are strongly correlated with their venous draining pattern, especially the presence of reflux into pial veins [5]. The Borden classification and Cognard classification, which are based on the characteristics of draining veins, represent the two most widely used systems. Low-grade fistulas (Borden I; Cognard I, IIa) are considered begin, and aggressive symptoms such as intracranial hemorrhage, intracranial hypertension, focal neurologic deficits, and seizures are found more commonly with higher-grade lesions [6]. Tentorial DAVFs, accounting for almost 4–12% of all DAVFs [7], are often associated with more aggressive neurological behaviors [8].

CCF is defined as abnormal communication between carotid arteries and the cavernous sinus, while it usually presents with ocular and orbital symptoms [9]. The clinical manifestations of DAVF around the cavernous sinus or other types of anterior cranial fossa DAVF with anterior venous drainage could be similar to those of CCF. However, tentorial DAVF with posterior drainage seldom presents with ocular symptoms initially.

For falcotentorial DAVF, a kind of tentorial DAVF, the most common draining veins are the straight sinus, vein of Galen and torcular [10]. In this case, we reported a rare case manifested with ophthalmic complaints caused by a falcotentorial DAVF due to its posterior-to-anterior draining pattern, which caused left-side increased intraocular pressure. It is difficult to promptly diagnose tentorial DAVF causing ocular symptoms at the very beginning of clinical practice.

The goal of treatment is complete and permanent obliteration of abnormal arteriovenous shunts. Endovascular embolization (i.e., transarterial, transvenous, and direct cavernous sinus routes) with embolic agents is proven to be safe and effective and has been extensively used in clinical practice [11–13]. In this case, the fistula was successfully occluded by the transarterial approach. Transvenous embolization is feasible for high-flow fistulas or cases with multiple, small, and tortuous feeders. In addition, open surgery and radiosurgery are alternative options if endovascular therapy is exhausted. Owing to the complex vascular anatomy, preoperative angiographic evaluation is essential before surgical and endovascular treatment. The different therapeutic strategies can be used based on the angioarchitecture, clinical presentation, location, and operator preference.

## Conclusion

Cerebral vascular diseases presenting with ophthalmic complaints are not necessarily associated with CCF or cavernous sinus DAVF. Some specific subtypes of DAVF with anterior venous drainage should also be considered. Differential diagnosis and definitive treatment are mandatory to avoid a delayed diagnosis and irreversible symptoms.

## Data Availability

Not applicable.

## Abbreviations

CCF: Carotid–cavernous fistula
DAVF: Dural arteriovenous fistulas
DSA: Digital subtraction angiography
MMA: Middle meningeal artery
PCA: Posterior cerebral artery
